# Action Generation Adapted to Low-Level and High-Level Robot-Object Interaction States

**DOI:** 10.3389/fnbot.2019.00056

**Published:** 2019-07-24

**Authors:** Carlos Maestre, Ghanim Mukhtar, Christophe Gonzales, Stephane Doncieux

**Affiliations:** ^1^UMR 7222, ISIR, Sorbonne Université and CNRS, Paris, France; ^2^UMR 7606, LIP6, Sorbonne Université and CNRS, Paris, France

**Keywords:** skill building, action generation, learning from demonstration, affordances, motor control, state, Bayesian inference, closed-loop

## Abstract

Our daily environments are complex, composed of objects with different features. These features can be categorized into low-level features, e.g., an object position or temperature, and high-level features resulting from a pre-processing of low-level features for decision purposes, e.g., a binary value saying if it is too hot to be grasped. Besides, our environments are dynamic, i.e., object states can change at any moment. Therefore, robots performing tasks in these environments must have the capacity to (i) identify the next action to execute based on the available low-level and high-level object states, and (ii) dynamically adapt their actions to state changes. We introduce a method named Interaction State-based Skill Learning (IS^2^L), which builds skills to solve tasks in realistic environments. A skill is a Bayesian Network that infers actions composed of a sequence of movements of the robot's end-effector, which locally adapt to spatio-temporal perturbations using a dynamical system. In the current paper, an external agent performs one or more kinesthetic demonstrations of an action generating a dataset of high-level and low-level states of the robot and the environment objects. First, the method transforms each interaction to represent (i) the relationship between the robot and the object and (ii) the next robot end-effector movement to perform at consecutive instants of time. Then, the skill is built, i.e., the Bayesian network is learned. While generating an action this skill relies on the robot and object states to infer the next movement to execute. This movement selection gets inspired by a type of predictive models for action selection usually called affordances. The main contribution of this paper is combining the main features of dynamical systems and affordances in a unique method to build skills that solve tasks in realistic scenarios. More precisely, combining the low-level movement generation of the dynamical systems, to adapt to local perturbations, with the next movement selection simultaneously based on high-level and low-level states. This contribution was assessed in three experiments in realistic environments using both high-level and low-level states. The built skills solved the respective tasks relying on both types of states, and adapting to external perturbations.

## 1. Introduction

Autonomous robots are expected to help us in our daily tasks. In tasks involving objects, these robots must perform actions that result in a change of the object states, e.g., changing an object position or increasing its temperature. Therefore, in order to solve these tasks a robot must possess a repertoire of actions producing expected changes, called *effects*. The variability of environments to perform a task makes hard for a robot designer to foresee all the possible situations the robot can face and predefine an action for each case. For example, during the last DARPA Robotic Challenge (Atkeson et al., 2018) several robots failed to perform a trial due to the execution of built-in actions under incorrect circumstances. Therefore, it is plausible to think that a robot must develop its own behavioral capacities through interactions with the environment and learn when to use them.

Based on this principle, in our previous work (Maestre et al., 2017) we developed a method for a robot to build its own skills. A simulated Baxter robot endowed with our method executed an exploration of a static environment learning to push an object to specific positions of the environment. More precisely, the robot built a sensorimotor skill that generated actions producing different effects in the object states. We defined *state* as a feature that is relevant for a task, e.g., the object position; sensorimotor skill, or just *skill*, as the process transforming robot and object states into robot motor commands; and *action* as a sequence of movements of the robot's end-effector inferred in a closed-loop by a skill. The skill was implemented as a Bayesian Network (BN), a graphical representation of dependencies for probabilistic reasoning (Pearl, 1988). The exploration of the environment performed by the robot generated a dataset of robot-object interactions, henceforth *interactions*, that was used to learn both the network structure and the CPDs. The results showed that it was possible to build the skill through simple interactions with the object. However, both the exploration and the environment were constrained: the exploration was performed using predefined movements of a fix length in a two-dimensional environment that could be only modified by the robot actions. Besides, the generated push actions produced rough trajectories. Therefore, it was necessary to scale up the method for a robot to solve tasks in more realistic environments in which: (i) the robot environment is three-dimensional and dynamic, i.e., the object states can change at any moment independently of the robot actions; (ii) the task requires the use of complex actions, i.e., pick-and-place an object; (iii) action selection also implies abstract states, e.g., an object is hot or grasped; and (iv) actions are continuous and must adapt to changes in the environment.

In the current paper we introduce an extension of our previous method named Interaction State-based Skill Learning (IS^2^L), which builds skills to reproduce effects on objects in realistic environments. The main features of the method are threefold: first, the method generates continuous actions in three-dimensional environments that locally adapt to spatio-temporal perturbations (Gribovskaya et al., 2011), similarly to Khansari-Zadeh and Billard (2014) and Paraschos et al. (2017). Spatial perturbations are those related to changes of the spatial values of a state. For example, changes of the initial position of the robot's end-effector w.r.t. the object position before the execution of an action, or changes of the object position during the execution. Temporal perturbations are those related to a change of the duration of an action, i.e., if the robot's end-effector gets stuck or delayed during the execution of the action. The adaptation to these spatio-temporal perturbations is performed through a data augmentation of the available interactions using a dynamical system called *diffeomorphism* (Perrin and Schlehuber-Caissier, 2016). This method proposes to apply a deformation to the motion space that generates a vector field converging to the expected trajectory to execute. And thus a robot action can recover from a perturbation executing the motion described by the vector field.

Second, a skill built with our method generates actions simultaneously relying on the low-level and high-level states of both the robot and the environment objects during the interactions. *High-level states* are those representing higher level concepts related to action selection, e.g., an object color or shape (Montesano et al., 2008). *Low-level states* are those related to the execution of an action, e.g., an object position (Calinon et al., 2010). An interaction is represented as a sequence of high-level and low-level states and the next robot movement to perform at different instants of time. Therefore, the action generation consists in, given an effect to reproduce and both types of states, choosing the next movement to perform among all the possible ones. Namely, the BN selects the movement with highest posterior probability. This movement selection gets inspired by a type of predictive models for action selection usually called *affordances* (Jamone et al., 2016; Zech et al., 2017). An affordance is initially defined as the actions an agent can afford to execute through direct perception of an object (Gibson, 1966, 1986). In robotics, it has been defined as the acquired relation of applying an action on an object to obtain an effect (Sahin et al., 2007).

Third, the method builds complex trajectories using *imitation learning* (Billard and Calinon, 2016), in which an external agent performs one or more kinesthetic demonstrations of an action generating a dataset of low-level states of the robot and the environment objects.

The main contribution of this paper is combining the main features of dynamical systems and affordances into a single method to build skills that solve tasks in realistic scenarios. More precisely, combining the low-level movement generation of the dynamical systems, to adapt to local perturbations, with the next action selection simultaneously based on high-level and low-level states.

Three experiments, of increasing complexity, were executed to assess the feasibility of the method to generate skills using both low-level and high-level states. In the first experiment, the robot pushed an object to a final position in different mazes, only using the object position (low-level states). In the second experiment, the robot grasped a croissant and released it in a pan using as information the object positions (low-level states) and if the croissant was grasped at an instant of time (high-level state). Finally, in the third experiment the robot had to heat the croissant to a certain temperature (high-level state) turning a stove on and off pressing a button (high-level state). These experiments were directly performed by a physical Baxter robot, showing that the method is able to generate skills to solve tasks in realistic environments.

The remainder of this paper is organized as follows. Section 2 describes the background and works related to our method. Section 3 describes IS^2^L. Section 4 describes the experiments and obtained results. Section 5 provides some conclusions to this study and identifies some possible future research lines.

## 2. Related Work

There is certainly a lack of works in the robotics literature combining action selection (using high-level states) with adaptive action execution (using low-level states). To the best of the authors' knowledge, Kroemer et al. (2012) is the only work combining these features. In this work, a pouring task experiment is executed, in which a robotic arm *grasps* a watering can and *pours* water into a glass. The main objective of this experiment is to use affordance knowledge to learn predictive models mapping subparts of objects to motion primitives based on direct perception. The main different between our work and the one presented by Kroemer et al. consists in that they focus on the low-level features of an object, i.e., its shape acquired using a point cloud, to select the next action to apply; whereas our work uses a simpler low-level representation of the object, i.e., its location represented as a position, combined with other high-level object features for the action selection. A positive aspect of their work is that the method directly uses a sensor information as input, providing richer object information, which can help to generate accurate interactions with the objects. However, in order to handle high-level features the method should be combined with another method working in parallel, adding a relevant complexity to the symtem.

The remainder of the section introduces works related to either selecting the next action to perform (based on predictive models) or building a skill to reproduce an action (based on imitation learning and motor control techniques) using either anthropomorphic robots or robotics arms.

### 2.1. Selecting the Next Action To Perform

In the works introduced in this section action selection either relies on affordance knowledge or are based on non-linear mappings from raw images to robot motor actions. Actions are usually considered as built-in knowledge, externally tailored by a designer, and they are executed in an open-loop. These works are only robust to spatial perturbations before the execution of an action, i.e., to the object position, not adapting the action to spatial and/or temporal perturbations during its execution. This offline spatial adaptation is usually externally hard-coded by the experiment designer. This low adaptation capability can result in the inability to scale up the executed experiments to realistic setups.

The works depicted in Table 1 are categorized based on the classification available in Jamone et al. (2016). The relevant categories for the current work are *Pioneering works* representing those first studies where the initial insights to learn the relation between objects and actions were identified; *Representing the effects* is the category with more related works, including IS^2^L, and extends the previous action-object relations to take into account the corresponding effect; *Multi-object interaction* represents affordances among several objects; and finally *Multi-step prediction* represents the use of affordances in high-level task planners to solve complex tasks.

**Table 1:** Comparison of actions used within the affordance literature, where *represents ambiguous information.

**Type**	**Publication**	**Affordance learning method**	**AA**	**OffSP**	**OnSP**	**TP**	**PA**	**RA**
Pioneering works	Krotkov, 1995	–	–	No	No	No	Yes	Poke
	May et al., 2007	–	–	No	No	No	No	Random
	Metta and Fitzpatrick, 2003, Fitzpatrick and Metta, 2003	–	–	Object position	No	No	Yes	Tap
	Fitzpatrick et al., 2003	PI	–	Object position	No	No	Yes	Tap
	Stoytchev, 2005	DT	–	Object position	No	No	No	Random
Representing the effects	Demiris and Dearden, 2005	BN	–	Object position	No	No	No	Random
	Hart et al., 2005	DRN	–	Object position	No	No	Yes	Grasp
	Lopes et al., 2007, Montesano et al., 2008, Osório et al., 2010	BN	–	Object position	No	No	Yes	Grasp, Tap, Touch
	Ugur et al., 2009, 2011	SVM	–	Object position	No	No	Yes	Push
	Ridge et al., 2010	NN	–	No	No	No	Yes	Push
	Kopicki et al., 2011	LWPR	–	Object position	No	No	No	Push
	Ugur et al., 2012, 2015a	SVM	–	Object position	No	No	No	Grasp, Hit, Drop, Tap
	Mugan and Kuipers, 2012	DBN	–	Object position	No	No	Yes	Grasp
	Hermans et al., 2013	SVR	–	Object position and orientation	No	No	Yes	Push
	Finn et al., 2016, Finn and Levine, 2017	LSTM	–	Object position and orientation	No	No	No	Push
	Ebert et al., 2017	LSTM	–	Object position and orientation	No	No	Yes, No	Lift, Push
	Hangl et al., 2016	MMR	–	Object position and orientation	No	No	Yes	Push, Flip
	Chavez-Garcia et al., 2017	GBN	–	Object position	No	No	Yes	Push, Grasp
	This work	BN	LH	Object position	Yes	Yes	No	Push, Grasp, Press
Multi-object interaction	Jain and Inamura, 2013	BN	–	Object position	No	No	Yes	Push, Pull
	Goncalves et al., 2014	BN	–	No*	No	No	Yes	Tap, Push, Pull
	Dehban et al., 2016, 2017	DA	–	No*	No	No	Yes	Push, Pull
Multi-step predictions	Omrčen et al., 2008, Krüger et al., 2011	NN	–	Object position and orientation	No	No	Yes	Poke, Push, Grasp
	Ugur et al., 2015b, Ugur and Piater, 2015	SVM	–	Object position	No	No	Yes	Pick, Place, Poke, Stack
	Antunes et al., 2016	BN	–	No*	No	No	Yes	Grasp, Release, Pull

*Works are categorized based on the classification available in Jamone et al. (2016) (see column Type). They are described based on the following features: Affordance learning method, AA, Action adaptation; OffSP, Offline Spatial Perturbation; OnSP, Online Spatial Perturbation; TP, Temporal Perturbation; BA, Built-in actions; RA, Repertoire of actions. The affordance learning methods are PI, Probabilistic Inference; DT, Decision Tree; BN, Bayesian Network; DRN, Relational Dependency Network; SVM, Support Vector Machine; NN, Neural Network; LWPR, Locally Weighted Projection Regression; DBN, Dynamic Bayesian Network; SVR, Support Vector Regression; LSTM, Long Short-term Memory; MMR, Maximum Margin Regression; GBN, Gaussian Bayesian Network; DA, Denoisy autoencoder*.

The goal of the pioneering works (Krotkov, 1995; Fitzpatrick and Metta, 2003; Metta and Fitzpatrick, 2003; May et al., 2007) was identifying affordances observing the result obtained when applying an action on an object, e.g., rollability. Posterior works (Fitzpatrick et al., 2003; Stoytchev, 2005) made the first attempts to learn the relation between the action and the obtained result, trying to choose the best action to reproduce it. However, actions and effects were very simple. In contrast, the works representing the effects focus on learning an inverse model to reproduce a previously observed effect on an object. Dearden and Demiris (2005), Demiris and Dearden (2005), and Hart et al. (2005) are the first works to propose representing the forward and inverse models using Bayesian Networks (BN) in this context, used to play imitation games. Inspired by the previous works, Lopes et al. (2007), Montesano et al. (2008), Osório et al. (2010), and Chavez-Garcia et al. (2016) define an affordance as a BN representing the relation between action, object and effect. They provide built-in grasp, tap, and touch actions to also play imitation games. Similarly, other works also use built-in actions using different methods to learn affordances, as classification techniques (Ugur et al., 2009, 2011; Hermans et al., 2013), regression methods (Kopicki et al., 2011; Hermans et al., 2013; Hangl et al., 2016), neural networks (Ridge et al., 2010), dynamical BN (Mugan and Kuipers, 2012), among others. Multi-object interactions has gathered many research attention during the last years, mainly focused on the use of tools to reproduce effects on objects. Jain and Inamura (2011), Jain and Inamura (2013), Goncalves et al. (2014), and Goncalves et al. (2014) use a BN to model affordances to push and pull objects using tools with different features, whereas Dehban et al. (2016) and Dehban et al. (2017) use Denoising Autoencoders. Conversely to tool use, Szedmak et al. (2014) proposes to model the interactions of 83 objects with different features assisted by a human expert. In the previous works a repertoire of built-in actions was available for the affordance learning. Nevertheless, a couple of works by Ugur and his collaborators built this repertoire beforehand (Ugur et al., 2012, 2015a). In these works a built-in generic swipe action is available, which executes a trajectory of a robot's end-effector from a fixed initial position to the position of a close object. Therefore, for different object positions different trajectories are built. Nevertheless, the shape of these trajectories does not differ much among them, because of the use of the same heuristic to generate them. Other works in the same vein are Finn et al. (2016), Finn and Levine (2017), and Ebert et al. (2017), which use a deep learning technique called convolutional LSTM (Hochreiter and Schmidhuber, 1997) in order to predict the visual output of an action. These works build a repertoire of continuous push actions based on an exploration performing thousands of interactions of a robotic arm with a set of objects (see Wong, 2016 for a recent survey about applying deep learning techniques in robotics).

### 2.2. Reproducing an Action

A robot can learn from demonstration all the actions required to reach a task goal. This section presents some of the most relevant works building skills, also called motion primitives, reproducing an action from one or more demonstrations. In Table 2 there is a comparison of these works. The variables selected for the comparison represent the capability of a skill to adapt to low-level (L) and high-level states (H), together with the main features studied within the motor control literature: mechanisms to be robust to spatio-temporal low-level perturbations, the stability of a motion primitive, the number of examples needed for the learning, and the combination of different primitives to reproduce an unseen action.

**Table 2:** Comparison of methods generating adaptive skills.

**Type**	**Publication**	**MP learning method**	**AA**	**Spatial perturbation**	**Temporal perturbation**	**TD**	**St**	**NE**	**C**
Trajectory-based	Ijspeert et al., 2002, Ijspeert et al., 2013	DMP	L	Final position	No	Yes	Yes	1	No
	Pastor et al., 2009, Kober et al., 2010	DMP	L	Final position and velocity	No	Yes	Yes	1	No
	Muelling et al., 2013	MoMP	L	Final position and velocity	No	Yes	Yes	1	Yes
	Paraschos et al., 2013, Paraschos et al., 2017	ProMP	L	All positions and velocities	Yes	No	Yes	M	Yes
	Perrin and Schlehuber-Caissier, 2016	Diffeomorphism	L	Final position	Yes	No	Yes	1	No
State-based	Calinon et al., 2007	GMR-DS	L	No	Yes	No	No	M	–
	Calinon et al., 2010, Calinon et al., 2011	HMM + GMR	L	Final position	Yes	No	No	M	–
	Khansari-Zadeh and Billard, 2011, Khansari-Zadeh and Billard, 2014, Kim et al., 2014	SEDS	L	Final position	Yes	No	Yes	M	–
	Calinon, 2016	TP-GMM	L	All positions and orientations	Yes	No	Yes	M	–
	This work	IS^2^L	HL	All positions	Yes	No	No	M	Yes

*Works are categorized based on the classification available in Paraschos et al. (2017) (see column Type). They are described based on the following features: MP, Motion primitive; AA, Action adaptation; SP, Spatial perturbation; TP, Temporal perturbation; TD, Time-dependency; St, Stable; NE, Number of examples; C, Combination of MPs*.

Paraschos categorizes motion primitives as *trajectory-based representations*, which typically use time as the driving force of the movement requiring simple controllers, and *state-based representations*, which do not require the knowledge of a time step but often need to use more complex, non-linear policies. Paraschos et al. (2017, p. 2). On the one hand, trajectory-based primitives are based on dynamical systems representing motion as time-independent functions. The principal disadvantage of dynamical systems is that they do not ensure the stability of the system. In order to address this issue, an external stabilizer based on time to generate stable motion is used (e.g., DMPs, Ijspeert et al., 2002, 2013; Pastor et al., 2009; Muelling et al., 2013). Therefore, actions are always executed following a specific time frame. A more recent approach called ProMP (Paraschos et al., 2013, 2017) avoids the previous constraint by generating time-independent stable primitives.

On the other hand, state-based motion primitives are time-independent by definition, in which the states use continuous values and are represented by Gaussian functions. For a specific position of the robot's end-effector, weights are computed using Hidden Markov Models (HMM) to identify the next state based on the current state. Once the state is available, the motion is computed using Gaussian Mixture Regression (GMR). The initial works (Calinon et al., 2007, 2010, 2011) do not generate stable actions, but it has been solved in posterior studies by a method called Stable Estimator of Dynamical Systems (SEDS) (Khansari-Zadeh and Billard, 2011, 2014; Kim et al., 2014), which ensures stability through a computation of Lyapunov candidates (Slotine and Li, 1991). However, SEDS can only handle spatial perturbations at the final position of the demonstrated trajectories. This feature is improved in Calinon (2016) handling spatial perturbations at any position of the trajectory, through the generation of a set of waypoints around the trajectory with different reference frames.

As aforementioned, works in the literature focus on either selecting the next action to perform a task based on high-level states using predefined or constrained actions; or in the reproduction with local adaptation of the trajectories of a complex action using low-level object states. Therefore, the skills built by IS^2^L are unique to infer actions with local adaptation simultaneously based on both types of states.

## 3. Interaction State-Based Skill Learning (IS^2^L)

**Figure 1:**
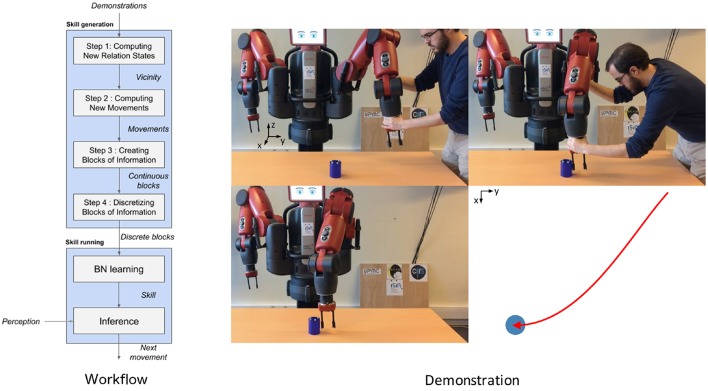
On the left, a flowchart of the steps of the method. On the right, an initial kinesthetic demonstration of a trajectory *pushing* an object. At the top-left corner, the setup of the experiment. At the top-right corner, the demonstration performed by a co-author of this paper. At the bottom-left, the object was pushed certain distance and orientation. At the bottom-right, top-view graphical representation of the action. The red arrow represents the demonstrated trajectory, and the blue circle represents the final position of the object. Although the reference frame of the setup is located in the base of the robot, in order to facilitate the visual comprehension of the setup the reference frames are depicted in different places.

This section explains the method Interaction State-based Skill Learning (IS^2^L). Given several examples of a robot performing an action, i.e., producing a specific effect, on an object, the method creates a skill that generates actions reproducing the effect on the object. These actions can cope with local changes in the position of the object. At the left side of Figure 1 a flowchart of the steps of the method is available. The method is based on the interactions between the robot's end-effector performing the action and the object. An interaction represents a sequence of the robot and object states during a period of time. More precisely, at each instant of time the high-level states of the robot and the object, and the low-level state representing the relative position of the object with respect to the robot, called *relation state*, are represented.

Each interaction is composed as a sequence of (i) high-level states of the robot and the object, and (ii) the low-level state representing the relative position of the object with respect to the robot, called *relation state*, at different instants of time. Robot actions and object effects represent the difference of these states between two consecutive instants of time. The main advantage of this approach is that the method does not build skills reproducing an interaction in a specific scenario. These skills use the most relevant information during the interactions, i.e., high-level states and relation states, to infer actions under similar robot-object interactions with local adaptation to perturbations.

A skill is a BN that, given an effect to reproduce and a relation state, infers the next robot movement to perform (see the next sections for further details). In order to simultaneously handle high-level and low-level states, the BN uses discrete values, although the inferred robot action is continuous. In the current paper, the set of actions a robot can perform is composed of *push, grasp, release, set* and *press*.

### 3.1. Initial Available Information

Some available information is needed to execute the method. First, interactions must represent the relevant states to perform different actions on objects. IS^2^L relies on a previous developmental stage identifying these states that E. J. Gibson calls *differentiation* (Gibson, 2000, 2003), which is out of the scope of our work (a recent and relevant approach is available in Jonschkowski and Brock, 2015; Jonschkowski et al., 2017).

Second, some a priori information is needed to build a skill. A BN is a graphical representation of dependencies for probabilistic reasoning, in which the nodes represent *random variables* and the lack of arcs represent *conditional independence relationships* between the variables (Pearl, 1988). More precisely, a BN is a directed acyclic graph, i.e., a collection of nodes or vertices joined by directed edges without directed cycles. Besides the structure, which provides qualitative information about the probabilistic dependencies between the variables, a BN also encodes quantitative information about the strength of these dependencies through Conditional Probabilistic Distributions (CPDs). In the current work, the structure represents the knowledge that an interaction is based on the relative position of the end-effector and an object, and the actual values of the interaction are stored as CPDs. In our previous work (Maestre et al., 2017) a simulated Baxter robot executed an exploration of a static environment identifying the BN structure and CPDs to *push* an object in different directions. The results demonstrated that the BN structure is task- and environment-agnostic. Therefore, in the current paper the structure is provided. And thus building a skill consists in learning the correct CPDs to reproduce an effect. Second, in our previous work we also identified a generic discretization configuration to discretise the relation states (explained at the end of section 3.2).

Finally, a dataset of interaction demonstrations, 𝔻, must be available to build skills (at the right side of Figure 1, a demonstration of a *push* action). For the aforementioned list of possible actions, the low-level states represent at each instant of time the end-effector position, *x*_*t*_, and the object position, *y*_*t*_; whereas the high-level states represent at each instant of time the discrete gripper openness (open/closed), *G*_*t*_, and object high-level states, *H*_*t*_, representing different object features^1^. Therefore, an interaction, Υ_*xygh*_, is represented as:

(1) $$\begin{array}{l} \begin{array}{c} x_{t}=\text{end effector position}\\ G_{t}=\text{gripper state}\\ y_{t}=\text{object position}\\ H_{t}=\text{high-level object states}\\ \Upsilon_{xygh}=\left\{\left(x_{0},y_{0},G_{0},h_{0}\right),\ldots ,\left(x_{T},y_{T},G_{T},h_{T}\right)\right\} \end{array} \end{array}$$

An effect is defined as an expected variation of the object states, $\hat{\Lambda f}$, in between two instants of time, *t* and *t-1*, and it is associated to a label, *e*. The effect can be reproduced multiple times, and thus it is not related to any specific instant of time. In the current work the expected variation can be related to either a variation of the object position or a variation of the high-level object states:

$$\begin{array}{l} e\equiv \hat{\Lambda f}=y_{t}-y_{t-1}\vee H_{t}-H_{t-1} \end{array}$$

where the subscript *t* represents an instant of time.

Therefore, a dataset of interactions is represented as:

(2) $$𝔻=(e,\{\Upsilon_{xygh}^{k}\})$$

where *k* represents one of the *K* interactions available.

### 3.2. Skill Generation

Once the dataset of interactions is available the method to build the skill starts, which is composed of two processes:

- *Dataset augmentation and transformation*: first, the dataset of interactions, 𝔻, is extended and transformed into a repertoire, *R*, of discrete *blocks* of information (section 3.2.1).
- *Skill building*: second, the skill is built based on the dataset of blocks, i.e., the CPDs of the BN are learned (section 3.2.2).

#### 3.2.1. Dataset Augmentation and Transformation

The dataset of interactions, 𝔻, represents one or more interactions producing an effect on an object. This sections explains the initial interaction augmentation and their posterior transformation into a sequence of blocks of information. A block of information, *B*, represents the relation of some high-level states to some low-level states at an instant of time to reproduce an effect on an object. More concretely, each block is a triple composed of (i) the relation state at an instant of time, δ, (ii) the high-level states of the robot and the object at that instant, *H*, and (iii) the next movement of the end-effector to execute reproducing an effect in an object, (Λ*x*_*t*_, Λ*g*_*t*_):

$$\begin{array}{l} B=\left(\delta ,H,\left(\Lambda x_{t},\Lambda G_{t}\right)\right) \end{array}$$

$$\begin{array}{l} R=\left\{B\right\} \end{array}$$

where Λ represents a difference of value of a variable between two instants of time, *t* and *t-1*.

Once *R* is available the CPDs can be learned, reproducing the same actions that were demonstrated and captured in 𝔻. However, with the current dataset if the robot faces an unobserved relation state, for example due to noise in the actuators of the robot or external forces, the BN would not be able to infer any movement. Namely, the skill is not yet robust to spatio-temporal perturbations.

It would be highly expensive to record interactions of the robot reproducing an effect from very similar relation states. Therefore, the method generates an augmentation of the blocks in 𝔻 adding*different but close* relation states. The approach is inspired from Calinon et al. (2010), where a set of Gaussians is computed along a demonstrated trajectory describing end-effector movements converging to the trajectory. IS^2^L computes a sampling of positions of the end-effector around the trajectory of the demonstrated interaction generating the new relations states, called *vicinity* (Step 1). Then, for each new relation state of the vicinity an end-effector movement is computed using a dynamical system (Step 2), and a new discrete block of information is stored into *R* (Steps 3 and 4).

**Figure 2:**
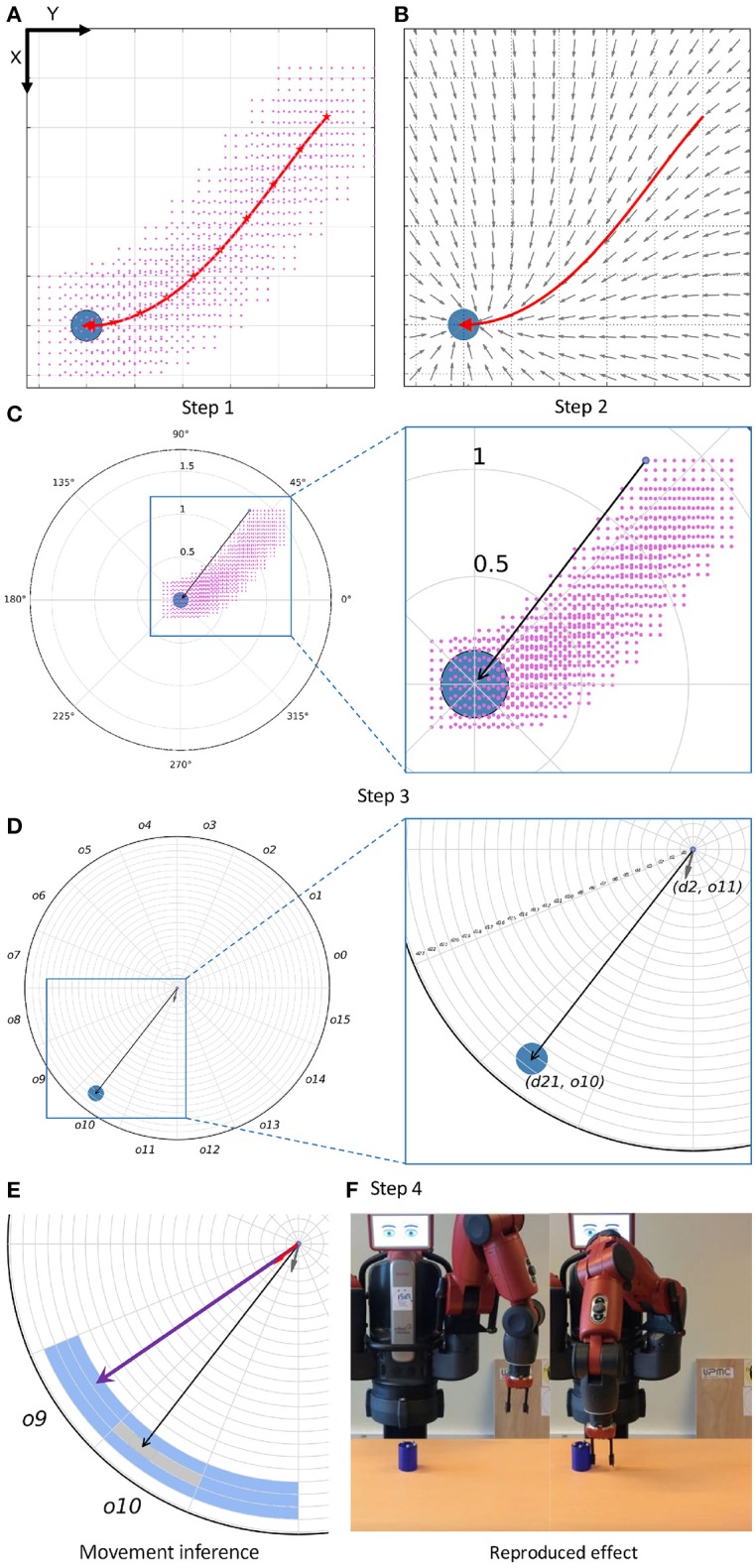
**(A)** Example of the computation of a vicinity. The trajectory is performed in the Cartesian X-Y plane, and the figure represents the top view of the setup. The trajectory is represented with a red arrow, and the object with a blue circle. The red stars represent the waypoints selected to compute the vicinity of the trajectory. For each of them, a set of end-effector positions is generated, represented by the pink points. **(B)** Example of the computation of movements from new positions of the end-effector. Each gray arrow represents the next movement, from a position, to be executed by the end-effector in order to reproduce the demonstrated effect. **(C)** Example of the computation of a continuous relation state (black arrow) a position of the vicinity of the trajectory (selected with a blue circle). **(D)** Example of the discretization of a block of information, i.e., discretizaton of both the continuous relation state and movement (gray arrow) into a *distance* and an *orientation*. In this case, the discrete relation state has values *d21* and *o10*, whereas the discrete movement has values *d2* and *o11*. **(E)** Example of the inference of a movement by the skill. A neighbor of the previous block of information is depicted, being its relation state represented as a purple vector (*d21, o9*) and its movement as a red vector (*d2, o9*). **(F)** Initial and final instants (from left to right) of the reproduction of the demonstrated effect.

*Step 1: Computing New Relation States Using a Vicinity*. A vicinity is computed for the trajectory of each demonstrated interaction. First, the trajectory is reduced to a set of equidistant waypoints (represented as red stars in the Step 1 of the Figure 2). The number of waypoints is computed based on the length of the trajectory. The higher the number of waypoints, the more precise the representation of the demonstration. However, a very high number of waypoints can affect the speed in which the BN infers a movement, because of the size of the CPDs. For each waypoint a vicinity is created, i.e., a sampling of unobserved end-effector positions. A vicinity is represented as a cubic grid centered in the waypoint with side size *Q*, and composed of *P* x *P* x *P* equidistant positions, *P* and *Q* being preset values. For each position of a vicinity, i.e., for each new end-effector position, a relation state is computed.

*Step 2: Computing End-effector Movements for the New Relation States*. Similarly, for each position of the vicinity the corresponding movement of the end-effector is computed using a *vector field*. This field generates a vector, i.e., a movement, for any position of the end-effector. End-effector positions close to the trajectory generate similar movements to the trajectory, whereas far end-effector positions generate movements less similar to those of the trajectory, mainly oriented to its end. Therefore, only those positions in the vicinity of the trajectory are relevant to reproduce an effect. An example of a vector field is depicted in the Step 2 of Figure 2.

In the current work, vector fields are generated using a dynamical system called *diffeomorphism* (Perrin and Schlehuber-Caissier, 2016). This method proposes to apply a deformation to the motion space in order to fit a simple trajectory to a demonstrated interaction trajectory. More precisely, the approach aims to minimize a defined distance between both trajectories using a diffeomorphic matching algorithm. This dynamical system has a parameter to compute the tendency to reproduce the demonstrated trajectory. As the possible actions generated by our method share similar features, i.e., they are based on interactions of a gripper and an object, the parameter value is empirically preset.

*Step 3: Creating The Blocks of Information*. Once both the new relation states and the robot movements are available, the new blocks of information are created. To that end, the high-level states related to each waypoint of the trajectory, *W*, are correlated to the relation states and movements created in the corresponding vicinity, *V*. Therefore, for each position of *V* a new block is created, composed of (i) the robot and object high-level states at waypoint *W*, (ii) the relation state computed from that position and (iii) the robot movement computed from the same position.

*Step 4: Discretizing the Blocks of Information*. The BN needs discrete information to infer a discrete movement. Therefore, each block of information is discretized before being stored into *R*. As the high-level information is already discrete, only the relation states and the movements are discretized. Both are vectors defined in a three-dimensional Cartesian space, composed of a *distance*, an *orientation* and an *inclination*. However, vector discretization in the Cartesian coordinates is complex, due to the range of each axis is [−∞, ∞]. For this reason these vectors are transformed to *spherical coordinates* before being discretized. A vector in spherical coordinates is composed of a *distance*, with range [0, ∞], an *orientation*, with range, [−π, π] and an *inclination*, with range [0, π]. In the current work, the range of the *distance* is limited to the maximal reach distance of the robot's end-effector, i.e., 0.5. The values for the *orientation* and *inclination* are predefined based on experience, i.e., their ranges are divided into a preset number of *bins* of the same size. However, the distance size is task-agnostic because it determines the accuracy of the movements. For the available set of actions the distance of each movement is computed w.r.t. to the distance between two positions in the vicinity:

(3) $$minimal\;distance\;bin\;size=\frac{Q}{P-1}$$

#### 3.2.2. Building the Skill

Once the discrete repertoire of blocks, *R*, is available, the skill is built. As aforementioned, a skill, ϕ, is a BN that infers discrete movements, Λ*X*, to reproduce a discrete effect, *E*, on an object. Each movement is generated w.r.t to both the discrete relation state, δ, and the discrete high-level robot and object states, *H*, at certain instant of time. In parallel to the inference of the movement, the method also infers the next open/close action of the end-effector based on the robot high-level state, if the skill is related to the *grasp* action. Movement and gripper actions are independently inferred:

(4) $$\begin{array}{l} \left(\Lambda X_{t},\Lambda G_{t})=\phi (E,\delta ,H\right)\\ \;\Lambda X_{t}=\underset{{}_{\Lambda X_{t}}}{\text{arg max}}\;P(\Lambda X_{t}|E,\delta ,H)\\ \;\Lambda G_{t}=\underset{{}_{\Lambda G_{t}}}{\text{arg max}}\;P(\Lambda G_{t}|E,\delta ,H) \end{array}$$

A discrete movement is described using three discrete values, i.e., the distance, the orientation and the inclination:

$$\begin{array}{l} \Lambda X_{t}=\left(\Lambda_{dist}X_{t},\Lambda_{orien}X_{t},\Lambda_{inclin}X_{t}\right) \end{array}$$

Although it is possible that there is a weak dependency among these values, in order to speed up the computation of a movement we consider that these values are independent. And thus the inference of a movement consists in the individual inference of each one of them:

(5) $$\begin{array}{l} \Lambda X_{t}=(\underset{{}_{{\text{Λ}}_{dist}X_{t}}}{\text{arg max}}\;P(\Lambda_{dist}X_{t}|E,\delta ,H),\\ \;\underset{{}_{{\text{Λ}}_{orien}X_{t}}}{\text{arg max}}\;P(\Lambda_{orien}X_{t}|E,\delta ,H),\\ \;\underset{{}_{{\text{Λ}}_{inclin}X_{t}}}{\text{arg max}}\;P(\Lambda_{inclin}X_{t}|E,\delta ,H)) \end{array}$$

**Figure 3:**
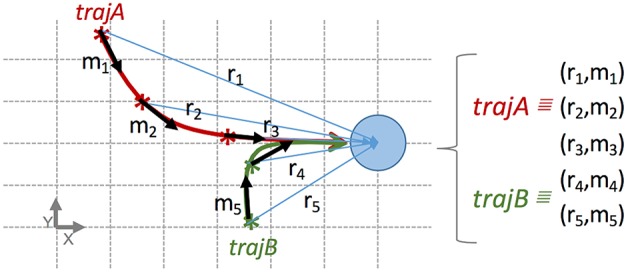
Example transforming two interactions into blocks of information stored in the same repertoire, used to build a skill reproducing the same effect from two different initial relation states (in order to facilitate the comprehension, only low-level states are used). Each block is represented as a tuple (relation state, movement), e.g., r_1_, m_1_. The figure represents a top-view in a table-top scenario similar to the demonstration in Figure 1. The blue circle represents an object. The robot pushes the object to the right from two initial positions of its end-effector. More precisely, the trajectory of two interactions, A (red) and B (green), reproduce the same effect from two different initial relation states. Each trajectory is split up into few blocks stored in the same dataset. In this case, the trajectory A is split up in blocks 1, 2, and 3; and the trajectory B in blocks 4 and 5. All blocks are mutually independent from temporal and spatial point of views. Namely, with this dataset our method is able to build a skill inferring the next movement to push the object to the right from 5 different relation states.

A relevant feature of our method is that skills directly combine information from different demonstrations. More precisely, the discrete repertoire of blocks, *R*, can contain information of one or more interactions, i.e., they have been computed based on trajectories of different demonstrations. The blocks of information generated from the different trajectories are stored into the same repertoire of blocks. And thus the related skill can infer movements combining information from different demonstrations (see Figure 3). It may happen that for the same relation state more than one movement have been stored in the same repertoire. These cases are directly handled by the probability distributions of the BN, calculating different probabilities for each movement.

### 3.3. Reproducing an Effect on an Object

When a skill is available, the inference and execution of each movement is performed within a *perception-action cycle* (Kugler and Turvey, 1987; Warren, 1988). In a continuous loop, the perceptual information acquired by the robot's sensors is transformed into high-level and relation states and provided as input to the BN, which infers a movement. Then, the movement is executed by the robot using its inverse kinematic model. This execution generates a displacement of the position of the robot's end-effector, which can modify the robot's environment. If the effect has not been reproduced, or a maximum number of movements executed, a new iteration of the cycle is executed.

It may happen, depending on the vicinity parameters, that while reproducing an effect the end-effector moves to a position whose relation state is not stored into the repertoire of blocks, and thus the skill would not infer any movement and the effect would not be reproduced. Instead of identifying a task-dependent discretization configuration to cover all the possible relation states, movements are computed as the mean value of a set of relation states, Ξ. This set consists of the *nearest neighbors* relation states of the current relation state, including itself. For each dimension of the vector state (the distance *d*, the orientation *o* and the inclination *c*) the neighbors are the previous and the next relation bins based on the discretization configuration:

$$\begin{array}{l} mean\;relation\;state\;=\;[(\sum_{a=i-N}^{i+N}d_{a})/(N*2)\\ \;+1,(\sum_{u=i-N}^{i+N}o_{u})/(N*2)+1,(\sum_{q=i-N}^{i+N}c_{q})/(N*2)+1] \end{array}$$

where *f, g, h* represents the number of the current bin, and *N* represents the number of neighbors at each side of the current bin. An example of this computation only using a distance and an orientation is available in the movement inference of Figure 2. In this example the current relation state is *d21* and *o10*. For one neighbor, *N=1*, the ranges of nearest neighbors would be [*d20, d21, d22*] for the *distance*, and [*o9, o10, o11*] for the *orientation*. And thus the computed mean relation state [(d20 + d21 + d22) / 3, (o9 + o10 + o11) / 3] would be used, together with the high-level states to infer the next movement.

Once a discrete movement has been inferred it is transformed into a continuous movement to be executed by the robot end-effector. This process simply selects the mid value of the range corresponding to each dimension composing the movement. For example, for the movement (*d2, o11*) the function computes the mid value for the bins *d2* and *o11*.

## 4. Experimental Framework

Three experiments, of increasing complexity, were executed to assess the feasibility of the method to generate skills using both low-level and high-level states (see Table 3). In the first experiment, the robot pushed an object to a final position in different mazes, only using the object position (low-level states). In the second experiment, the robot grasped a croissant and released it in a pan using as information the object positions (low-level states) and if the croissant was grasped at an instant of time (high-level state). Finally, in the third experiment the robot had to heat the croissant to a certain temperature (high-level state) turning a stove on and off pressing a button (high-level state).

**Table 3:** Skills, objects and object states used in the experiments (LL and HL stand for low-level and high-level states, respectively).

**ID**	**Experiment**	**Skills**	**Objects**	**Object LL**	**Object HL**	**Robot LL**	**Robot HL**
1	Solving a Maze	Push Set	Cylinder Cake	X		X	
2	Grasping a Croissant with Spatio−temporal Perturbations	Grasp Release	Croissant Pan Dish Button	X		X	X
3	Heating a Croissant	Grasp Release Press	Croissant Pan Dish Button	X	X	X	X

**Figure 4:**
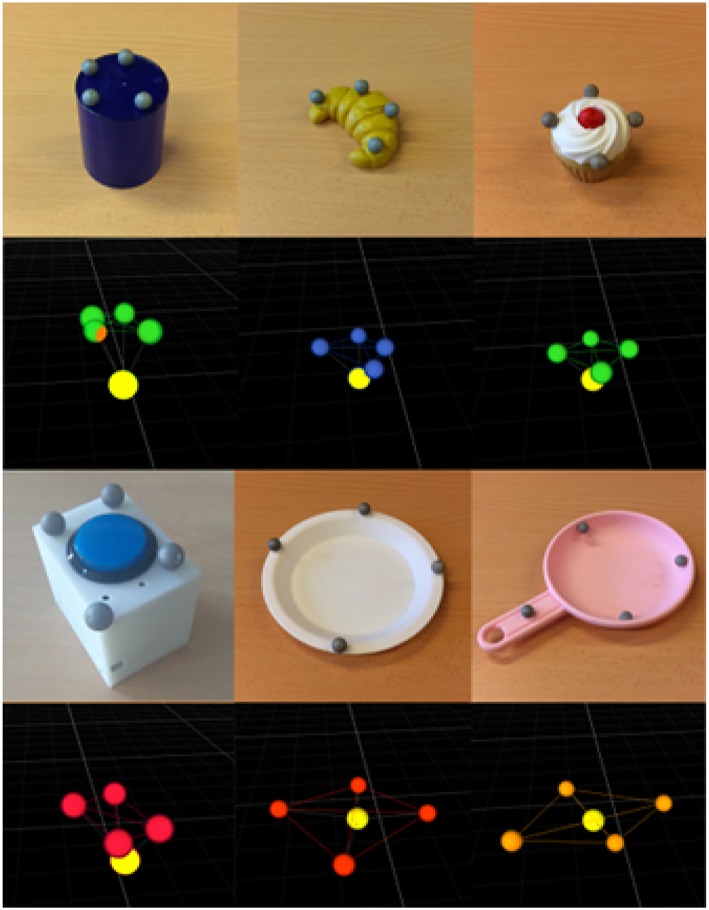
Set of objects used in the experiments, i.e., a cylinder, a croissant, a cake, a button, a dish and a pan, respectively. For each object a photo (at the top) and its representation captured by the OptiTrack system (at the bottom) are provided. The light yellow marker in each structure represents the center position of the object acquired by the robot.

Figure 4 shows the set of objects used for the experiments. The positions of the objects were acquired using an OptiTrack motion capture system^2^, composed of 4 cameras located at the ceiling, over the robotic setup. This system generated a virtual representation of each object, providing its center position, using markers located on it. The reference frame of the experimental setup was located at the base of the robot, and thus the object positions were relative to itself.

The validation of the method was performed on a physical Baxter robot. Each gripper of the robot had a different configuration: on the left gripper, the fingers of the gripper were in the farthest position, in order to grasp big objects; on the right gripper, the fingers were in a intermediate position, in order to grasp smaller objects. Both grippers had finger adapters in order to facilitate the corresponding targeted actions. The execution of the robot relied on ROS Indigo Igloo and our kinematic library^3^. Videos of the experiments are available online^4^.

### 4.1. A Priori Knowledge

**Figure 5:**
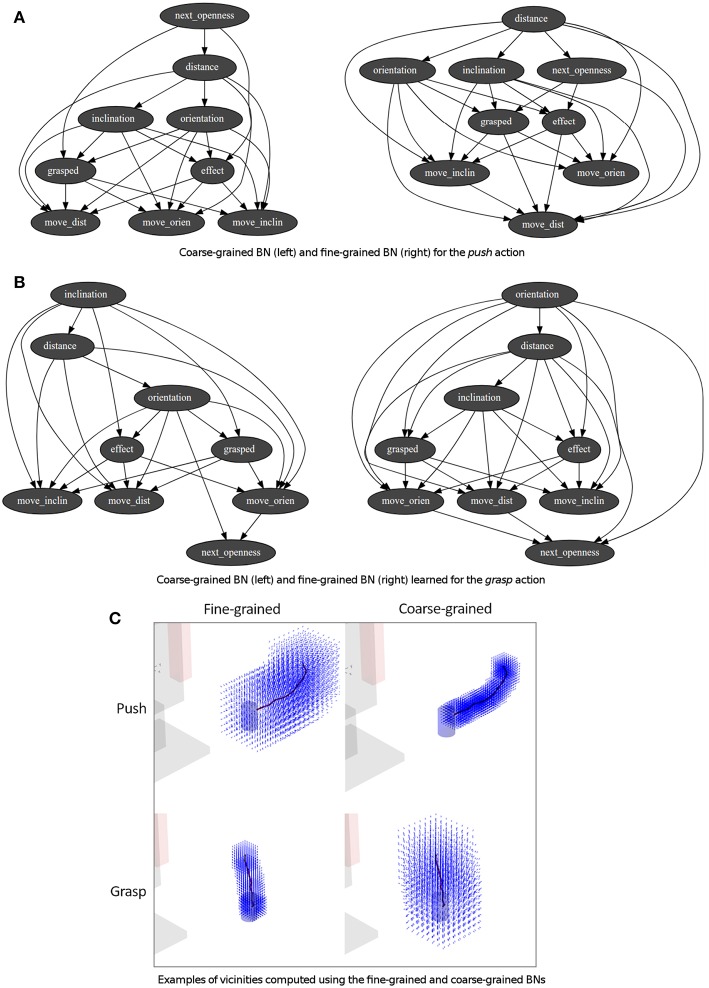
**(A,B)** BNs obtained from the demonstration to *push* and *grasp* an object. **(C)** Corresponding vicinities computed from the same demonstrations using the previous BNs.

One or more demonstrations were performed for each one of the skills used in the experiments, i.e., *push, set, grasp, release* and *press*. As aforementioned in the Step 1 of section 3.2.1, the accuracy of an action is based on the number of positions, *P*, and the size of the vicinity, *Q*, used to transform the demonstrations for the CPD learning. Based on these values two BNs with different levels of accuracy were learned using the available demonstrations (see Figure 5C): (i) a coarse-grained BN inferring bigger movements (around 6 cm) with *P* equal to 8 positions and *Q* equal to 40 cm, approaching the end-effector to the object; (ii) a fine-grained BN inferring small and more accurate movements (around 2.5 cm) with *P* equal to 7 positions and *Q* equal to 20 cm. These values were chosen based on experience. The fine-grained generator was used if the end-effector was close to an object (arbitrarily preset to 10 cm), whereas the coarse-grained generator was used in any other case. The gripper state was either *open* if its openness value was in its top half range, i.e., 50 or more over 100, or *closed* otherwise. Figures 5A,B show the structure of the learned BNs for the *push* and *grasp* skills. Some nodes represent the robot and object state before the execution of the movement: the nodes *distance, orientation, inclination* represent the relation state; and the node *grasped* represents if the object is grasped. The other nodes represent the movement to perform: the nodes *move_dist, move_orien* and *move_inclin* represent the end-effector movement; whereas *next_openness* represents the openness of the end-effector grippper.

For the discretization configuration, the distance had a range of [0, M], where M represents the longest distance of a movement of the robot, in this case 50 cm. This range was discretized in bins of the same size, which size is computed as in Equation 3. Finally, both the orientation and the inclination were split up in 16 bins of the same size.

### 4.2. Experiments

#### 4.2.1. Experiment 1: Solving a Maze

##### 4.2.1.1. Experimental Setup

**Figure 6:**
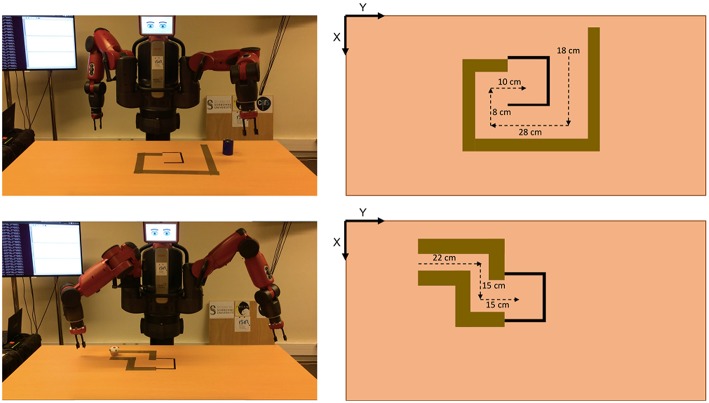
Setup of the mazes used in Experiment 1. At the top, for the first maze, and at the bottom, for the second maze. In both cases, from left to right, the physical setup and the expected distances to *push* the corresponding object in order to solve the maze.

A table of 180 × 80 × 75 cm of width, length, and height, respectively, was located in front of the Baxer robot (see Figure 6). The setup of this experiment consists of two mazes of different configurations. The objects to *push*, i.e., the cylinder for the first maze and the cake for the second maze, have different sizes, shapes and weights.

##### 4.2.1.2. Description

The task consisted in *pushing* an object through a maze to a final position. In these experiments the experiment designer chose the next action to execute and the distance to move the object, i.e., the effect to reproduce. Therefore, the goal of this experiment was to validate that the generated skills were able to reproduce an effect only relying on the object and gripper positions, i.e., low-level states. Besides, the experiments also validated the reproduction of different effects for the same skill, e.g., *pushing* an object to the right different distances.

In order to reproduce the sequence of actions different skills were demonstrated to the robot. First, a set of demonstrations were executed to *push* an object to the *left*, to the *right, close* to the robot, and *far* from the robot. Before executing each *push* action it is necessary to set the robot's end-effector on one side of the object, e.g., to *push* it to the right the end-effector must be located at the left of the object. Therefore, a set of demonstrations were executed to move the end-effector from the object to one of its sides (for example the C-D action on the top of Figure 8).

#### 4.2.2. Experiment 2: Grasping a Croissant With Spatio-Temporal Perturbations

##### 4.2.2.1. Experimental Setup

**Figure 7:**
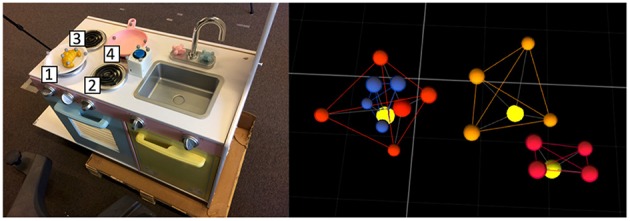
Setup used in Experiments 2 and 3. On the left, an image of the setup from the robot's point of view. On the right, example of the setup acquired by the motion capture system from the same point of view used for the image. The yellow markers represent the position of each object.

The scenario comprised a toy-like kitchen and other objects on it (see Figure 7). The kitchen, located in front of the robot, was composed of four stoves, a dish, a pan, a croissant and a switch button. The switch button turned on and off the stoves. This scenario was also used in the Experiment 3.

**Figure 8:**
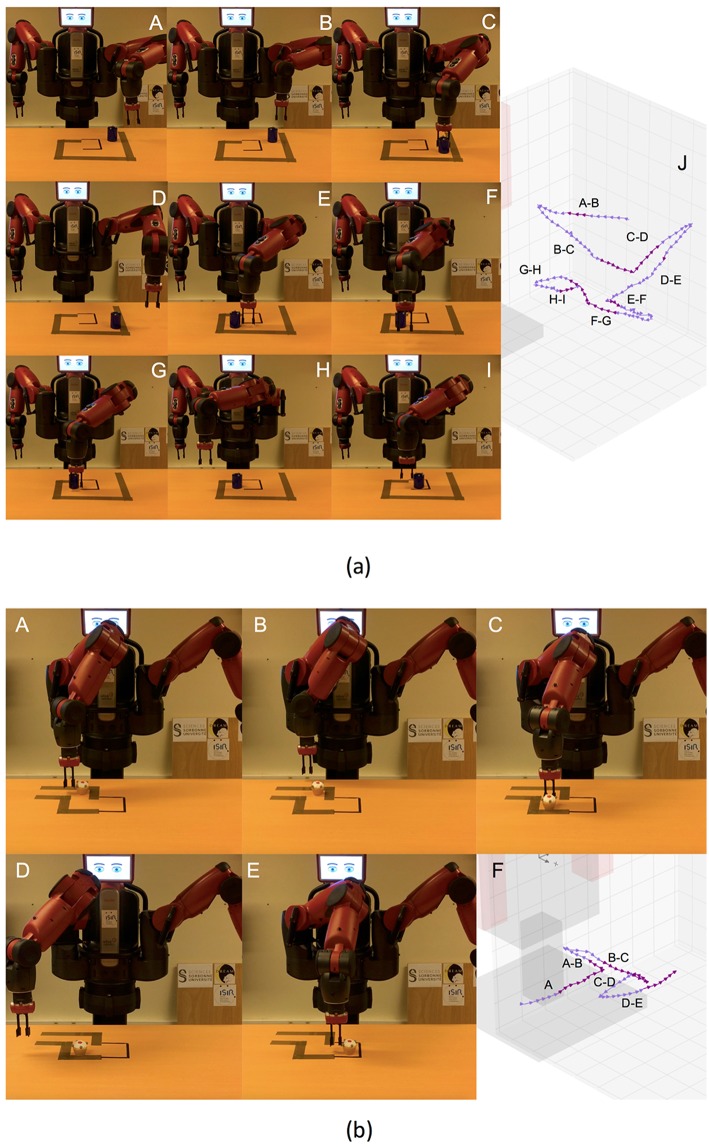
**(a)** Actions solving the first maze. From A to I, screenshots of the execution of the task. Finally, in J, virtual representation of the actions executed. **(b)** Actions solving the second maze. From A to E, screenshots of the execution of the task. Finally, in F, virtual representation of the actions executed.

##### 4.2.2.2. Description

**Figure 9:**
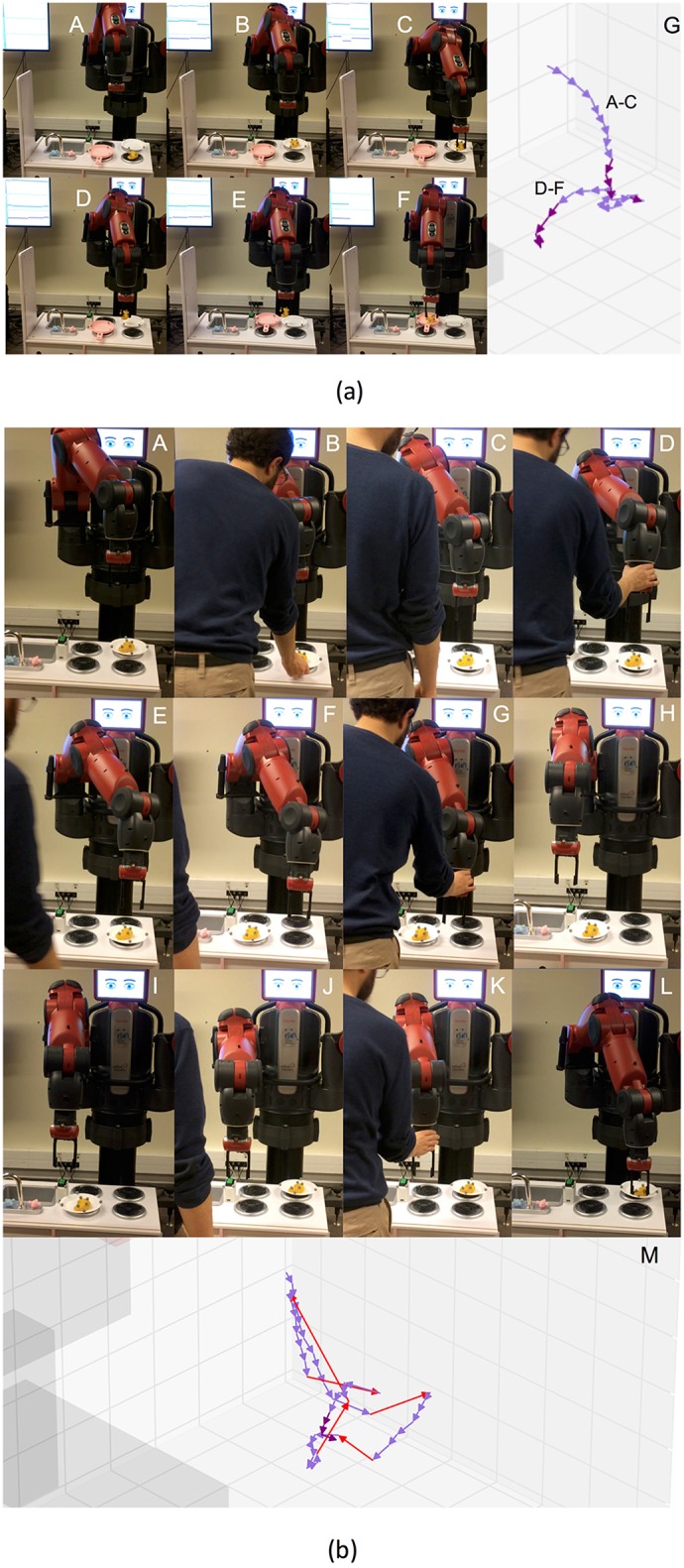
**(a)** Actions of the first spatio-temporal test, composed of *grasping* the croissant and *releasing* it into the pan. In this case, the spatial perturbation consists in changing the position of the pan during the *release* action. **(b)** Actions of the second spatio-temporal test, in which the robot tries to *grasp* the croissant. Both spatial and temporal perturbations are present, changing the position of the croissant, and externally moving the robot's end-effector, respectively. The orange arrows represent externally generated long movements of the end-effector. At the right side of the robot there is a screen showing the *distance, orientation* and *inclination* of the object w.r.t. the robot end-effector.

Two tests were carried out in order to validate reproducing effects using simultaneously both low-level features, i.e., the robot and object position, and high-level states, i.e., the *openness* state of the end-gripper. Also, the tests had to validate the robustness of the skills with respect to spatio-temporal perturbations of the low-level states. In the first test, the robot had to *grasp* a croissant and *release* it inside a pan. The position of the croissant changed during the *grasp* action whereas the pan position changed during the *release* action (see Figure 9a). The second test consisted in *grasping* the croissant. During the execution of the *grasp* action either the position of the end-effector was externally modified or the croissant position changed (see Figure 9b). In both tests the designer produced the perturbations.

#### 4.2.3. Experiment 3: Heating a Croissant

##### 4.2.3.1. Description

The objective of this experiment was to show that skills built by IS^2^L can be used to perform a multi-step task in a realistic scenario, simultaneously relying on high-level and low-level states of both the robot and the objects. The task consisted in heating a croissant in a pan until reaching a specific temperature. The high-level states of the objects were:

- Stove number 4 : *on* (red) or *off* (black).
- Croissant: *cold* (yellow), *hot* (salmon) or grasped (green).
- Button: *pressed* or *not pressed*.

**Figure 10:**
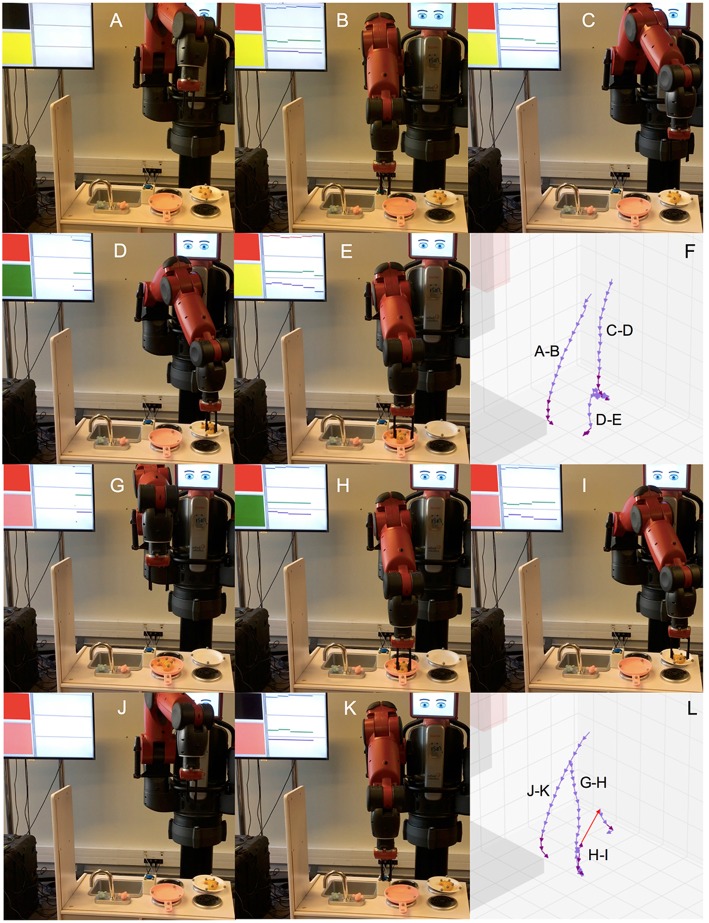
Actions of a successful execution of the task heating the croissant. From A to E, screenshots *pushing* the button, *grasping* the croissant, and *putting it* into the pan. In F, virtual representation of these actions. From G to K, screenshots *grasping* again the croissant, *putting it* back into the dish, and pressing the button. In L, virtual representation of these actions.

The different state colors were visually represented during the experiment in a screen next to the robot (see Figure 10). Initially, the stove was *off* , the button was *not pressed*, the *croissant* was *cold* and located in the dish, over the stove 1 (which was always off). If the croissant was in the pan, the pan was over the stove 4, and the stove was *on*, the temperature of the croissant changed from *cold* to *mid temperature* after few seconds; and from *mid temperature* to *high temperature* again after few seconds.

The available repertoire of actions were *pressing* the button, *grasping* the croissant, and *releasing* the croissant from the dish to the pan, and vice versa. Before the *grasp* and *press* actions the end-effector was randomly located over the setup, in a range of 20–40 cm of height, in order to show that actions can be inferred from different initial positions of the end-effector. The sequence of actions to reach the task goal was:

1. *Push* the button to turn the stoves on.
2. *Grasp* the croissant.
3. *Release* it into the pan.
4. When the croissant is hot *grasp* it again.
5. *Release* it back into the dish.
6. *Turn* the stove *off*.

In order to show that the skills built using our method can be directly combined with a task planner, before running the experiment a STRIPS planner with PDDL-like problem specification, called PyDDL^5^, was executed to compute the action order needed to solve the task. Then, the skills were built and associated to each action. Once the skills were available, a task manager executed them based on the action order and the object states. The task manager was also in charge of changing the colors of the screen representing the different object states.

### 4.3. Results

#### Experiment 1

The results obtained for both mazes are depicted in Figure 8. In both cases, the robot was able to solve the maze, showing a high precision for the *push* actions. Therefore, the skills built by IS^2^L can reproduce effects only based on the robot and object positions, i.e., low-level states. Besides, a skill could reproduce different results of the same effect, e.g., pushing different distances an object. Meaning these skills are task-agnostic and they can be used in different tasks.

At the top of the Figure, J shows the actions executed to solve the first maze. These actions are: (A-B) the robot *set* the end-effector *behind* the cylinder, (B-C) the robot *pushed* the cylinder *far*, (C-D) the robot *set* the end-effector *at the left* of the cylinder, (D-E) the robot *pushed* the cylinder to the *right*, (E-F) the robot *set* the end-effector *in front* of the cylinder, (F-G) the robot *pushed* the cylinder *close*, (G-H) the robot *set* the end-effector *at the right* of the cylinder, (F-G) the robot *pushed* the cylinder to the *left*. All the actions accurately reproduced the expected effect, except setting the arm *at the back* (A-B) and *in front* of the cylinder (E-F), due to reaching the kinematic limits of the right arm of the robot.

At the bottom of the Figure, F shows the actions executed to solve the second maze. These actions are: (A) the robot *pushed* the cylinder to the *right*, (A-B) the robot *set* the end-effector *at the back* of the cylinder, (B-C) the robot *pushed* the cylinder *far*, (C-D) the robot *set* the end-effector *at the right* of the cylinder, (D-E) the robot *pushed* the cylinder to the *right*, a different distance than A. Similarly, the less accurate actions (A and B) were those reaching the kinematic limits of the robot's arm.

#### Experiment 2

In both tests the *grasp* and *release* actions reproduced the expected effects using both high-level and low-level states. Besides, the skills were robust to the spatio-termporal perturbations, adapting the ongoing actions to the new object and end-effector positions.

Figure 9 shows the trajectories generated in the tests. At the top, the actions and the perturbations for the first test: (A-B) the robot tried to *grasp* the croissant, but its position changed from stove 3 to stove 1, (B-C) the robot adapted the actions and *grasped* it, (C-D) the robot executed the *release* action the croissant into the pan, (D-E) the pan position changed from the stove 4 to the stove 1, and the robot adapted its action, (E-F) the robot *released* the croissant in the pan. The action A-B shows a curve of the action from the end-effector random initial position toward the croissant position, until the latter changes, and a brusque change of direction appears. Similarly, the action C-D follows a trajectory from the croissant position to the pan position. Then, there is an abrupt change in the action direction when the pan position is changed.

At the bottom of the Figure, the actions and the perturbations for the second test: (A-B) from a random initial position over the kitchen the end-effector moved toward the croissant position until its moved from stove 1 to the stove 3, (B-D) the robot action adapted to the new position moving the end-effector far from the robot until the end-effector position was moved farthest than the croissant position, (D-E) the end-effector moved back to the croissant position until the croissant was moved to the stove 4, (E-H) the action again adapted moving toward the right until the end-effector was moved closed to the stove 1, (H-K) again the action adapted to the new end-effector position and moved toward the stove 3 whereas the end-effector was located on top of it, (K-L) finally the croissant is *grasped*. The trajectories of the arm are depicted, where the external changes in the position of the end-effector are identified as long orange arrows. It is very difficult to differentiate the actions due to the high number of changes produced.

#### Experiment 3

**Figure 11:**
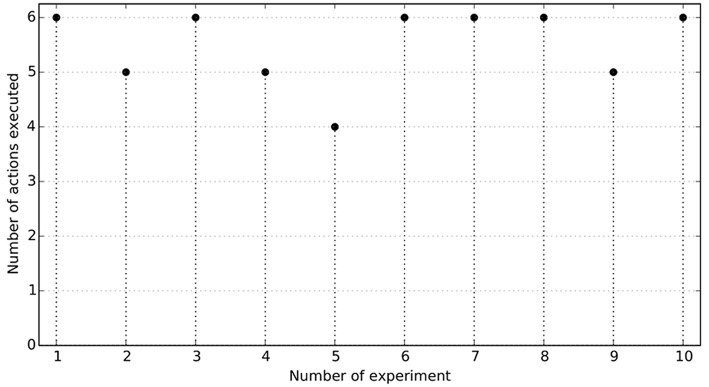
Results of 10 runs of the Experiment 3. The horizontal axis represents the number of action successfully executed of those listed in Experiment 3 (see section 4). A run is successful if the 6 actions are executed reproducing the expected effects.

Figure 11 shows the number of effects reproduced in 10 runs of the experiment. Six runs completely reproduced all the effects to solve the task, showing that the skills were able to solve a multi-step task in a realistic environment simultaneously using the low-level and high-level states of both the robots and the different objects within the scenario. Three times the robot was not able to properly *release* the croissant from the pan to the dish. The *release* actions mainly failed because these actions did not move the end-effector high enough and the markers of the croissant and the pan touched each other, displacing the pan and the dish, sometimes making the croissant fall from the end-effector. In one occasion the robot *grasped* the croissant from one of its extremes and this felt back to the dish. Figure 10 shows the actions of a successful execution of the task heating the croissant: (A-B) from a random initial position the robot *pressed* the button turning the stove 3 on, (C-D) from another random position the robot *grasped* the croissant, (D-E) the croissant was *released* in the pan, (G-H) from another random initial position the *grasp* action is executed once the croissant is hot, i.e., when the color in the screen changes from yellow to salmon, (I-J) the croissant is *released* back in the dish, (J-K) from another initial position the stove is turn off after the button is *pressed*. In general, the actions *pressing* the button were quite accurate. Also, the *grasping* actions were quite robust. The first *release* action (D-E) initially moved the end-effector up a high distance from the dish position, avoiding touching the dish markers. However, in the second *release* action, from the pan to the dish, the movements were lower, producing the objects to touch each other. New demonstrations showing the second *release* action with a higher height would generate a higher release action, importantly improving the success ratio of the experiment.

It is relevant to mention that just after the D-E action, and although the croissant was cold, we forced the task planner to execute the *grasping* action G-H. However, the skill was not able to infer any movement because it was built with the croissant temperature state as *hot*. Few seconds later when this state was reached the *grasp* action started.

## 5. Discussion and Conclusions

In the current paper we introduced a method named Interaction State-based Skill Learning (IS^2^L) that builds skills to reproduce effects on objects in realistic environments. These environments are three-dimensional and dynamic, i.e., the object states can change at any moment independently of the robot actions. Solving a task in these environments requires the use of complex actions, i.e., pick-and-place an object, that action selection also implies abstract states, e.g., an object is hot or grasped, and actions must be continuous and must adapt to changes in the environment. Therefore, a skill built with our method generates continuous actions that adapt to spatio-temporal perturbation, i.e., it generates in a closed-loop a sequence of movements of the robot's end-effector that adapts to changes of the object position. The skill was implemented as a Bayesian Network (BN). In our previous work (Maestre et al., 2017) we identified a task-agnostic BN structure useful for the action generation. Therefore, building the skill consists in learning the Conditional Probabilistic Distributions (CPDs) of the BN.

Before building the skill the experiment designer creates a dataset of one or more kinesthetic demonstrations of robot-object interactions producing an effect on an object. An interaction is represented as a sequence of high-level and low-level states and the next robot movement to perform at different instants of time. This dataset is used to learn the CPDs allowing the BN to infer the next robot movement for some specific high-level and low-level states. The inference of the this movement is inspired by the affordances action selection. Once this dataset is available the skill building starts, composing two processes: first, the demonstrated interactions are transformed into a repertoire of blocks of information, which represent the previous relationship between the high-level and low-level states and the robot movement. This repertoire is augmented with new relationships to make actions robust to perturbations, using a dynamical system called diffeomorphism. The BNs use discrete values for this relationship, and thus the augmented repertoire of blocks is discretized. In the second process, once this discrete repertoire is available a skill is built, i.e., the CPDs of the BN are learned. This skill infers discrete movements that are afterwards transformed into continuous movements using some simple heuristics.

The main contribution of this paper is a combination of the main features of dynamical systems and affordances in a unique method to build skills that solve tasks in realistic scenarios. More precisely, combining the low-level movement generation of the dynamical systems, to adapt to local perturbations, with the next movement selection simultaneously based on high-level and low-level states.

It is relevant to remark that for each experiment two BNs with different levels of accuracy were learned: a coarse-grained BN inferring bigger movements approaching the end-effector to the object, and a fine-grained BN inferring small and more accurate movements. This design decision was made to speed up the action execution, although only using the fine-grained BN would have been enough to generate the action. The minimal object distance to switch from the coarse-grained BN to the fine-grained one was set from experience, although it could be automatically identified based on a trial-and-error approach executing the same action few times (under similar conditions) and analyzing the quality of the obtained effect.

The learning of the BNs was fast and straightforward. However, depending on the size of the CPDs the movement inference was slow, and thus the movements of the robot although smooth were not as realistic as expected. Each BN structure is generated based on the corresponding dataset, and it represents different dependencies between the nodes based on the data available. The BN structures of the *push* and *grasp* skills identify the dependencies of the movement to perform with respect to the nodes describing the robot and object state before the execution of the movement, i.e., the relation state and the object being grasped. However, the dependency related to the *openness* of the gripper is only identified for the *grasp* skill, due to for the *push* skill the gripper remains closed.

Defining an action as a sequence of pairwise movements generated quite smooth trajectories. The interaction demonstrations were simple to generate, and to combine, providing a simple and flexible way of creating the initial dataset. The execution of these actions may produce some robot-object relation states unseen during the demonstrations, due to the noise generated by the robot joints. The method is robust to these situations thanks to the data augmentation, generating many different relation states around the demonstrations.

Although the quantity of a priori information needed to build the skills may look relevant, we have explained that in most cases the same values are useful for tasks sharing common abstract features, i.e., a gripper interacting with an object. Therefore, once a correct value is found for a variable this can be used for many different experiments.

A Baxter robot performed three experiments solving tasks of increasing complexity, using both low-level and high-level states. These experiments demonstrated that the method is able to generate skills useful for task solving in realistic environments. However, it is relevant to mention some aspects that can limit the scaling up of our method to more complex scenarios. The size of the CPDs grows exponentially when new states become relevant for a task, because of the *curse of dimensionality*. For example, if grasping objects of specific size, color and/or orientation. Similarly, performing tasks involving more than one object would generate the same dimensionality issue. This constraint has been already solved in Goncalves et al. (2014) reducing the dimensionality of the information provided to the BN using the Principal Component Analysis (PCA) technique. This approach would allow the BN to handle more information. However, the use of PCA or other dimensionality reduction techniques could complicate the identification of the proper BN structure. Another possible limitation is related to the complexity of the tasks. It would be necessary to test if tasks requiring high accuracy, e.g., putting a key in a keyhole and turning it, could be accomplished with the current task-agnostic parametrization of the method. Mainly with the proposed discretization configuration. Possibly new heuristics for the distance discretization would be necessary to generate more accurate robot movements.

Some possible improvements to the method would be to exploit all the information the dynamical system provides about the next movement, i.e., the *orientation, velocity* and *acceleration*. Currently, only the orientation is used by our method with a constant velocity. Extending the method to use the velocity and acceleration would result in more complex actions, e.g., *poke*. Also w.r.t. the dynamical system, adding areas to avoid to the generated vector fields, called *repellers*, would provide to the method an obstacle avoidance capacity, allowing the use of the method in more realistic environments.

## Author Contributions

CM was involved in the conception, design, and coding of the method. SD and CG are involved in the conception and design of the method. GM was involved in the coding of the method. CM, SD, CG, and GM wrote the paper.

### Conflict of Interest Statement

The authors declare that the research was conducted in the absence of any commercial or financial relationships that could be construed as a potential conflict of interest.

## Footnotes

^1^In the mathematical formalization uppercase variables represent states with discrete values, whereas lowercase variables represent states with continuous values.

^2^http://optitrack.com/

^3^https://github.com/cmaestre/baxter_kinematics

^4^https://www.youtube.com/playlist?list=PL2drYAFCMtzf4AC_ZRZjk8lNv2Zs9fh5Z

^5^https://github.com/garydoranjr/pyddl
